# Cell senescence, apoptosis and DNA damage cooperate in the remodeling processes accounting for heart morphogenesis

**DOI:** 10.1111/joa.12972

**Published:** 2019-03-15

**Authors:** Carlos I. Lorda‐Diez, Michelle E. Solis‐Mancilla, Cristina Sanchez‐Fernandez, Juan A. Garcia‐Porrero, Juan M. Hurle, Juan A. Montero

**Affiliations:** ^1^ Facultad de Medicina Departamento de Anatomía y Biología Celular and IDIVAL Universidad de Cantabria Santander Spain

**Keywords:** β‐galactosidase, cell death, cell senescence, H3K9me3, heart development, SASP, γH2AX

## Abstract

During embryonic development, organ morphogenesis requires major tissue rearrangements that are tightly regulated at the genetic level. A large number of studies performed in recent decades assigned a central role to programmed cell death for such morphogenetic tissue rearrangements that often sculpt the shape of embryonic organs. However, accumulating evidence indicates that far from being the only factor responsible for sculpting organ morphology, programmed cell death is accompanied by other tissue remodeling events that ensure the outcome of morphogenesis. In this regard, cell senescence has been recently associated with morphogenetic degenerative embryonic processes as an early tissue remodeling event in development of the limbs, kidney and inner ear. Here, we have explored cell senescence by monitoring β‐galactosidase activity during embryonic heart development where programmed cell death is believed to exert an important morphogenetic function. We report the occurrence of extensive cell senescence foci during heart morphogenesis. These foci overlap spatially and temporally with the areas of programmed cell death that are associated with remodeling of the outflow tract to build the roots of the great arteries and with the septation of cardiac cavities. qPCR analysis allowed us to identify a gene expression profile characteristic of the so‐called senescence secretory associated phenotype in the remodeling outflow tract of the embryonic heart. In addition, we confirmed local upregulation of numerous tumor suppressor genes including *p21, p53, p63, p73* and *Btg2*. Interestingly, the areas of cell senescence were also accompanied by intense lysosomal activation and non‐apoptotic DNA damage revealed by γH2AX immunolabeling. Considering the importance of sustained DNA damage as a triggering factor for cell senescence and apoptosis, we propose the coordinated contribution of DNA damage, senescence and apoptotic cell death to assure tissue remodeling in the developing vertebrate heart.

## Introduction

Cell death is a central mechanism of embryonic development associated with tissue differentiation and organ morphogenesis (Glücksmann, 1951; Saunders, 1966). Dying cells in embryonic systems usually present characteristic morphological features, which were taken as evidence of a common degenerative mechanism accounting for the dying process. The term ‘apoptosis’ was proposed to distinguish this type of cell death from degenerative processes resulting from direct cell insults involving rupture of cell membranes (Kerr et al. 1972). Apoptosis is characterized by massive cell densification and DNA fragmentation via specific endonucleases activated by a molecular cascade initiated either at the cell membrane (extrinsic pathway) or in the mitochondria (intrinsic pathway). In both apoptotic pathways, caspases, which are an evolutionary conserved family of proteases, exert a central function (Gross et al. 1999; Martinou & Green, 2001; Zamzami & Kroemer, 2001).

Studies of cell death in the developing *Caenorhabditis elegans* provided an important advance in understanding the genetic basis of the degenerative dying process, which appeared to be conserved among vertebrates (Stanfield & Horvitz, 2000). However, systematic analysis in vertebrate embryos, including genetic approaches, revealed that apoptosis is not the only degenerative event responsible for tissue remodeling. This is well illustrated by interdigital cell death that occurs during the formation of free digits in tetrapods, which is considered the most representative model of embryonic programmed tissue degeneration. Species with free digits, such as chick, mouse or human, exhibit massive apoptosis of the interdigital mesodermal tissue encompassed between the developing digit rays (Montero & Hurle, 2010). The intensity of interdigital apoptosis is much reduced in species with webbed digits, such as duck or bat. However, interdigit remodeling is not fully inhibited by genetic or chemical inhibition of caspases (Kuida et al. 1998; Chautan et al. 1999; Zuzarte‐Luis et al. 2007; Montero & Hurle, 2010) , suggesting that other degenerative events are also implicated in this morphogenetic process (Arakawa et al. 2017). Recent studies reported that interdigit remodeling involves an intense process of cell senescence (Lorda‐Diez et al. 2015). Furthermore, it appears that both cell senescence and apoptosis are preceded by previous events of massive DNA damage (Montero et al. 2016).

The developing heart provides a valuable model to characterize mechanisms responsible for tissue remodeling. Extensive work has revealed important areas of cell death in the vertebrate embryonic heart (Ojeda & Hurle, 1975; Okamoto & Satow, 1975; Pexieder, 1975; Hurle & Ojeda, 1979; Okamoto et al. 1979, 1981; Satow et al., 1981; Takeda et al., 1996; Poelmann et al. 1998; Watanabe et al. 1998; Ya et al. 1998; Poelmann & Gittenberger‐de Groot, 1999; Abdelwahid et al. 2002). Although differences in the intensity of cell death between avian and mammalian embryos have been reported, the overall pattern of cell death appears to be mostly conserved among different species (Sharma et al. 2004). The most intense and conserved foci of cell death appear during the morphogenesis of the outflow tract (OFT) and in the atrioventricular septum (AVS) (Manasek, 1969; Ojeda & Hurle, 1975; Pexieder, 1975; Hurle & Ojeda, 1979; Cheng et al. 2002; Sharma et al. 2004; Barbosky et al. 2006). Cell death in these areas has been related to the transition from single to dual circulation in the developing embryo (Watanabe et al. 2000, 2001). In the OFT, cell death is particularly relevant in the developing aorticopulmonary septum, semilunar valves and the myocardial layer of the conus (Manasek, 1969; Hurle & Ojeda, 1979; Kirby et al. 1983; Nakamura & Sumida, 1990; Poelmann et al. 1998; Watanabe et al. 1998; Cheng et al. 2002; Sharma et al. 2004; Barbosky et al. 2006). Cell death in the AV cushions and adjacent myocardium of the interventricular septum helps to establish the AV and interventricular septa (Pexieder, 1975; Wessels et al. 1996; Watanabe et al. 1998; Zhao & Rivkees, 2000; Cheng et al. 2002; Sharma et al. 2004; Barbosky et al. 2006). In addition to these well‐defined areas of cell death, scattered dying cells have been reported within the heart walls, which may have potential histogenetic significance (Fisher et al. 2000; Cheng et al. 2002; Sharma et al. 2004; Barbosky et al. 2006).

Upon DNA damage, an early key step of the DNA damage repair response (DDR) implies the recruitment to areas of DNA breaks of histone H2AX phosphorylated at serine 139 (termed γH2AX). This step allows the organization of a complex of DDR mediators that make γH2AX a commonly used marker for DNA damage events (Redon et al. 2011; Sharma et al. 2012). In this study, we sought to characterize the degenerative events that occur during heart morphogenesis. We show that there are well‐defined areas of cell senescence in the developing hearts of chick and mouse embryos that overlap with the known areas of apoptosis. In addition, we show that DNA damage, as detected by immunolabeling of γH2AX, is an accompanying feature of the zones of tissue regression.

## Material and methods

### Animal models

Embryonic hearts were obtained from Rhode Island chicken embryos from 4 to 8 incubation days (i.d.), corresponding with stages 23–33 of the Hamburger‐Hamilton criteria (from 23HH to 33HH) and C57BL6 mouse embryos from days 12.5 to 14.5 postcoitum (p.c.). The animal care and handling as well as all the experimental procedures were in accordance with the guidelines of the European Communities Council and the Spanish legislation, and were approved by the Service of Animal Health and Welfare of the Regional Government of Cantabria (Reference no. PI‐03‐18).

### β‐Gal activity

The β‐galactosidase activity assay was performed at pH 6 in vibratome sections of embryonic hearts after overnight (o/n) fixation in 4% glutaraldehyde following the recommendations of Debacq‐Chainiaux et al. (2009). Results presented in this work are representative of analysis of at least five independent hearts.

### *In situ* hybridization

*In situ* hybridizations were performed in microdissected heart fragments or in vibratome sections fixed using cold 4% paraformaldehyde (PFA) o/n. Specimens were treated with 10 μg mL^−1^ proteinase K for 20–30 min at 20 °C. Hybridization with digoxigenin‐labeled antisense RNA probes was performed at 68 °C. An alkaline phosphatase‐conjugated anti‐digoxigenin antibody (dilution 1 : 2000) was used (Roche). Reactions were developed with a BCIP/NBT substrate (Roche). The probes for chick cathepsin D were obtained by PCR.

### Antibodies, immunolabeling and confocal microscopy

Antibodies against cathepsin D (Santa Cruz Biotechnology), β‐galactosidase (Abcam), MF20 (Hybridoma bank), H3K9me3 (Abcam) and γH2AX (NOVUS) were employed for immunostaining. Counterstaining using fluorescent phalloidin (Sigma) was also performed. Appropriated fluorophore‐tagged secondary antibodies were used.

For immunolabeling, hearts were dissected free from embryos and fixed using cold 4% PFA o/n. Hearts were washed in phosphate‐buffered saline (PBS) and sectioned at 100 μm using a vibratome before immunostaining. Briefly, samples were permeabilized using several washes of 0.1% Triton‐PBS, incubated overnight in the primary antibody, washed with PBS, incubated for 2 h at room temperature (RT) using the secondary antibody, washed with PBS and finally prepared using a glycerol‐based mounting media for confocal analysis.

To obtain heart dissociated cells, microdissections of small squares of the OFT were dissected free under the microscope, fixed in PFA and carefully flattened with a coverslide. The slides were then deep frozen over dry ice to adhere the cells to the glass surface. Cells were permeabilized with 0.5% Triton X‐100 (90 min) before incubation with the primary antibody and, after several PBS washes, the cells were incubated with the secondary antibody.

After immunolabeling, the samples were examined with a laser confocal microscope (Zeiss LSM510) using Plan‐Neofluar 10×, 20× or Plan‐Apochromat 63× objectives and an argon ion laser (488 nm) to excite FITC fluorescence and a HeNe laser (543 nm) to excite TRITC. TIFF (RGB) images were transferred to Adobe photoshop software (Adobe Systems Inc.) for presentation. All images presented within each figure were identically adjusted for contrast, brightness and dynamic resolution. Each image in this work is representative of at least three independent experiments.

### TUNEL analysis of dying cells

Hearts were dissected free from chicken embryos and fixed using cold 4% PFA o/n; 100‐μm‐thick vibratome sections were obtained and specimens analyzed for apoptotic DNA fragmentation by the terminal deoxynucleotidyl transferase‐mediated dUTP‐TRIC nick end labeling (TUNEL) assay. We also employed TUNEL analysis on cell dissociates after immunolabeling. We followed the manufacturer's instructions for the *in situ* cell death detection kit (Roche). Counterstaining using fluorescent phalloidin (Sigma) was also performed. Samples were analyzed by confocal microscopy as described. Each image in this work is representative of at least three independent experiments.

### Real time quantitative PCR (qPCR) for gene expression analysis

Tissue fragments 400–500 μm thick were carefully microdissected from the OFT and the wall of the ventricles subjacent to the OFT of embryonic chick hearts at the desired stages. Total RNA was extracted using the NucleoSpin RNA kit (Macherey‐Nagel). First‐strand cDNA was synthesized using the High Capacity cDNA Reverse Transcription Kit (Life Technologies). The cDNA concentration was adjusted to 0.5 μg μL^−1^. SYBR Green‐based qPCR (Life Technologies) was performed using the Mx3005P system (Stratagene). A panel of the most characteristic components of the senescent secretome and cell cycle regulators was selected for our study. The samples obtained from the ventricular walls were employed for comparative purposes as a neutral non‐senescent region. *Gapdh* was chosen as the normalizer gene.

Mean values for fold changes were calculated. Expression level was evaluated relative to a calibrator according to the 2‐(ΔΔCt) equation. Each value represented the mean ± SEM of at least six independent samples obtained under the same conditions. Data were analyzed using Student's *t*‐test. Statistical significance was set at *P* < 0.05. qPCR specific primers for chick genes analyzed in this study are available upon request.

## Results

### Cell senescence forms well‐defined regions that overlap with areas of cell death in the developing heart

Recent studies have demonstrated that tissue remodeling during embryonic development involves cell senescence (Muñoz‐Espin et al. 2013; Storer et al. 2013; Muñoz‐Espin & Serrano, 2014; Lorda‐Diez et al. 2015). During development, the vertebrate heart undergoes dramatic morphogenetic changes that create a four‐chambered pumping organ from an initial tubular structure formed from modified blood vessels. Cell death is a major player in such tissue remodeling events (Hurle et al. 1977; Hurle & Ojeda, 1979; Sharma et al. 2004). By monitoring the activity of beta‐galactosidase at pH 6.0 (senescence‐associated β‐galactosidase, SA‐β‐Gal;), we detected intense areas of cell senescence during the period of septation of the developing avian heart (see Debacq‐Chainiaux et al. 2009). The period of intense cell senescence was extended between stage 26HH (5 i.d.) to 33HH (8 i.d.).

**Figure 1:**
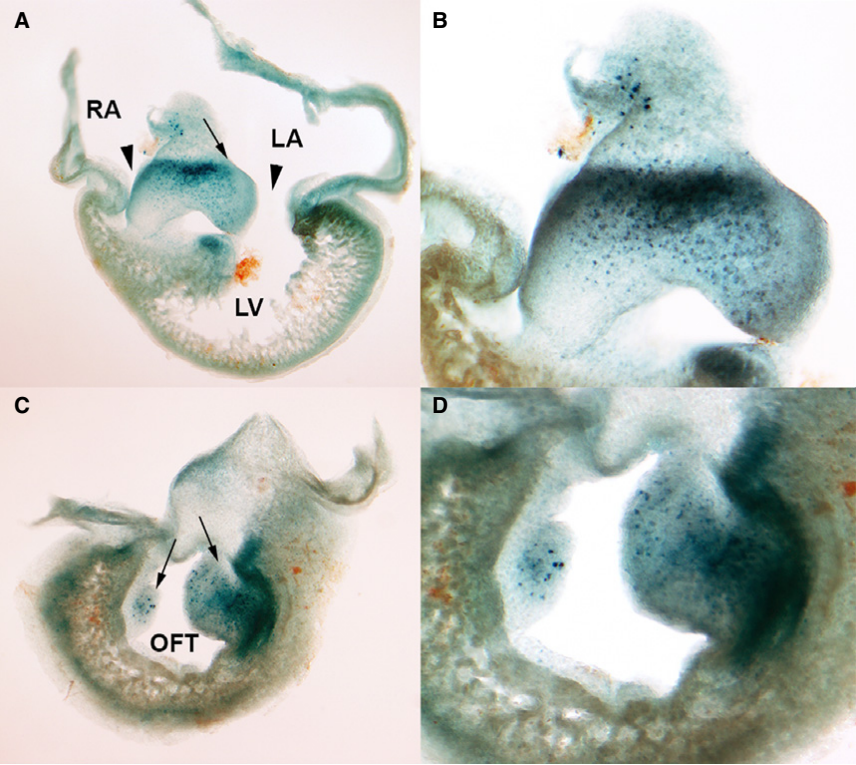
Areas of cell senescence in the developing heart of the chicken embryo at stage 26HH detected by SA‐β‐galactosidase activity staining. (A) Frontal section showing labeling in the atrioventricular cushion (arrow) and upper rim of the interventricular septum (IVS). (B) Detailed view of the interventricular septum in (A) to show intense labeling along the junction between the auricular and ventricular parts of the cushion. (C) Section across the developing outflow tract (OFT) showing positive labeling of the proximal outflow tract cushions (conal cushion). (D) Detailed view of the cushions. LA, left auricle; RA, right auricle.

**Figure 2:**
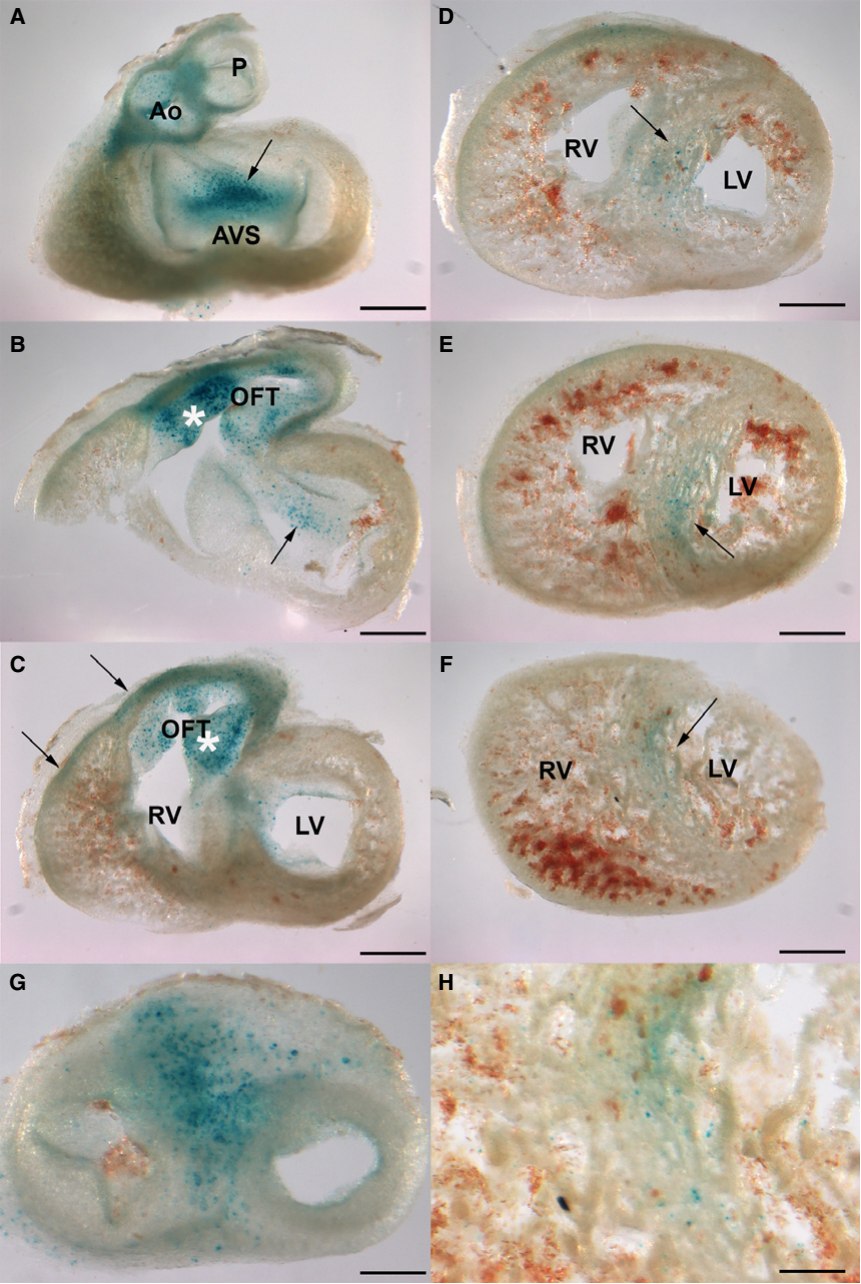
Areas of cell senescence in the developing heart of the chicken embryo at stage 29HH detected by SA‐β‐galactosidase activity staining. (A–F) Serial longitudinal vibratome sections showing the areas of SA‐β‐Gal activity. (A) Strong labeling in the atrioventricular septum (AVS) between the developing auriculo‐ventricular valves and in the valve region of the aorta (Ao) and pulmonary arteries (P). (B,C) Strong labeling in the outflow tract (OTF) including the cushions (*) and the ventral myocardial wall (arrows in C). (D–F) Successive sectioning through the ventricles showing positivity in the interventricular septum (arrow). (G,H) Detailed views of the root of the great arteries (G) and interventricular septum (H) illustrating positivity of the aorticopulmonary septum and interventricular septum. Scale bars: (A–F) 300 μm; (G–H) 100 μm.

Initial areas of cell senescence were appreciated at stage 26HH in the AV endocardial cushion (1A‐B) and in the OFT (Fig. 1C,D). By stages 29‐30HH (6–6.5 i.d.), cell senescence intensified at the level of the OFT (Fig. 2A–C,G). In this region, labeling was found both within the cushion tissue of the developing outflow septum and in the myocardial layer (Fig. 2A–C). Additional regions of intense SA‐β‐Gal positivity were the aorticopulmonary septum (Fig. 2G) and the atrioventricular developing septum (AVS; arrows in Fig. 2A–B). Finally, a number of cells with intense SA‐β‐Gal activity formed a well‐defined area in the upper part of the interventricular septum (IVS) (arrows in Fig. 2D–F and detailed view in Fig. 2H).

**Figure 3:**
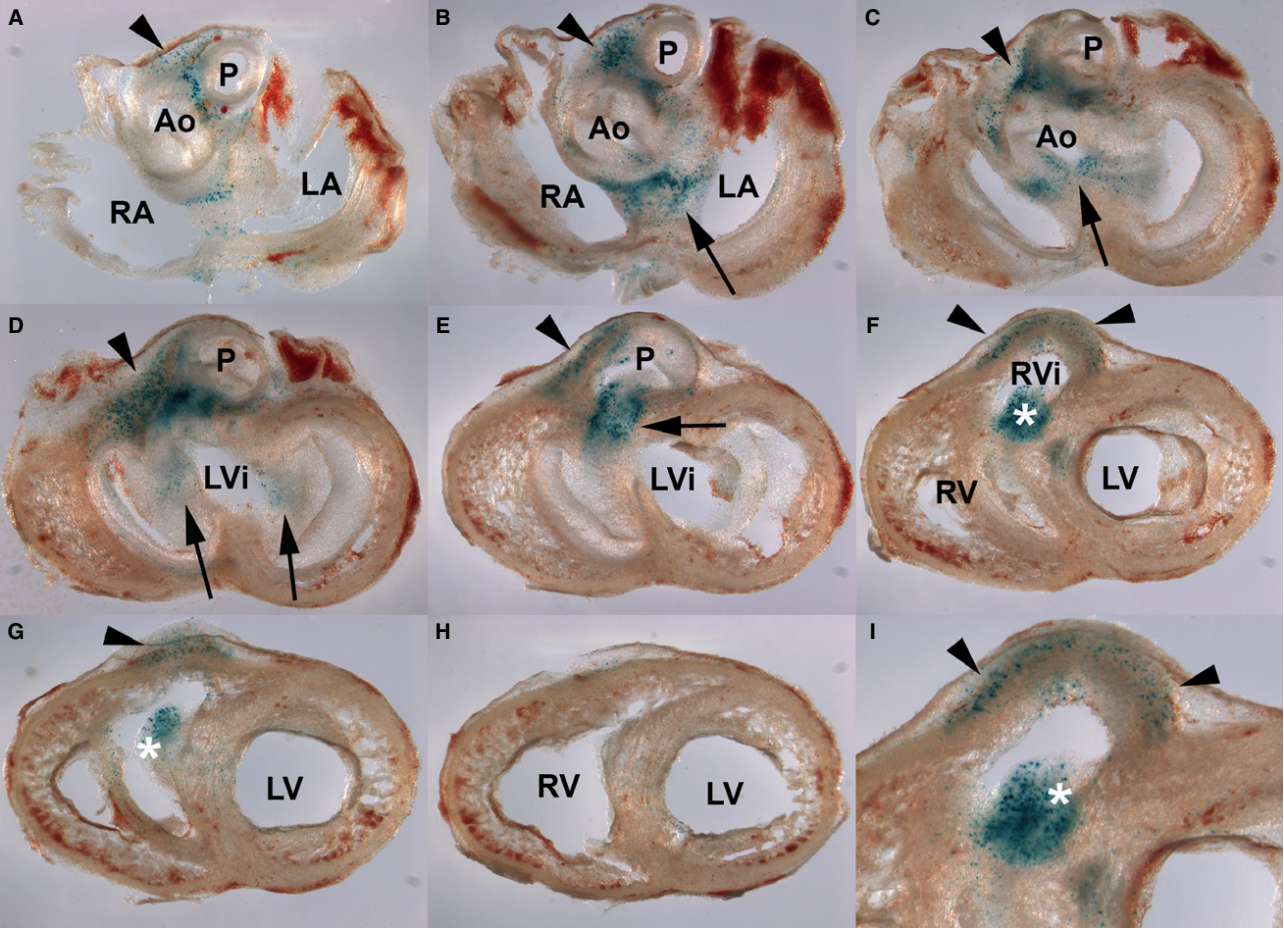
Areas of cell senescence in the developing heart of the chicken embryo at stage 31HH detected by SA‐β‐galactosidase activity staining. (A–H) Serial sections showing the areas of SA‐β‐Gal activity. As in previous stages, the highest positivity is observed in differentiating outflow tract, including the aorticopulmonary septum (arrowhead in A–E); the ventral surface of the right ventricular myocardial wall (arrowhead in F–G); and proximal OFT cushion (asterisk) and atrioventricular cushions (arrow in B). Note that the ventricular walls lack areas of positivity below the valvular apparatus (H). (I) Detailed view of (F) showing staining in the myocardial layer and proximal cushion of the outflow tract (asterisk). P, pulmonary artery; Ao, Aorta; RA, right atrium; LA, left atrium; LV, left ventricle; RV, right ventricle; LVI, left ventricle infundibulum; RVi, right ventricle infundibulum.

By stage 31HH, SA‐β‐Gal activity was still detected within the OFT, especially at the level of the aorticopulmonary septum (Fig. 3). Important labeling was also detected within the OFT myocardium, especially in the ventral surface (arrowheads in Fig. 3A–E), which extended into the infundibulum surface of the right ventricle (arrowheads in Fig. 3F,G and detailed view in Fig. 3I). SA‐β‐Gal labeling was also detected within the proximal OFT endocardial cushions (asterisk in Fig. 3F,G,I) and maturing AV septum (arrows in Fig. 3C–E), where AV valves are specified.

In subsequent stages, the zones of cell senescence became progressively reduced, and by stage 33HH (8 i.d.), only two SA‐β‐Gal‐positive domains were noticeable, namely, the aortopulmonary septum and the upper part of the interventricular septum (see Fig. 8A,C).

**Figure 4:**
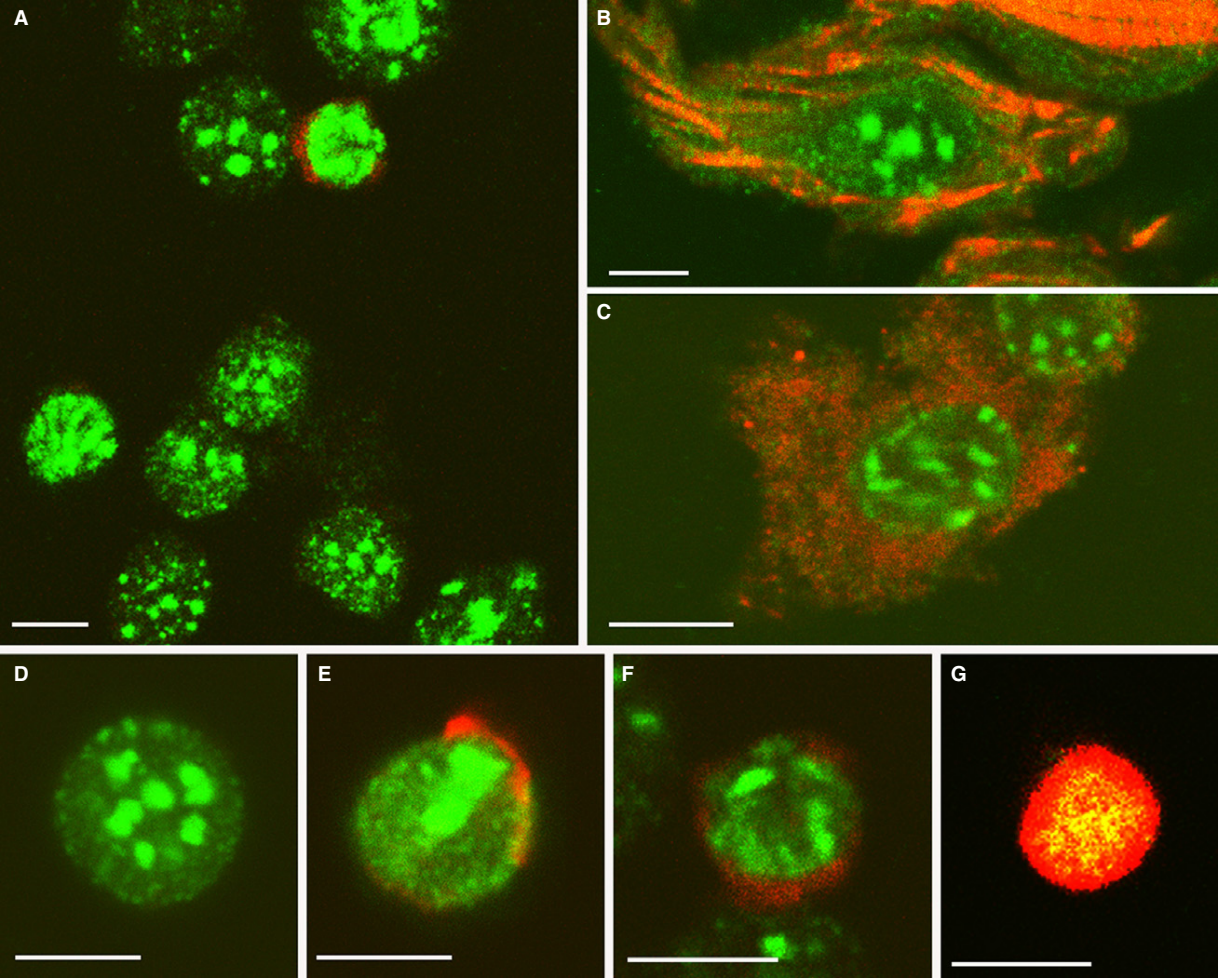
Enriched H3K9me3 nuclear immunolabeling in the remodeling OFT of the embryonic heart. (A,B) H3K9me3 foci (green) in cell dissociates of the outflow tract in combination with TUNEL assay (red in A) or phalloidin staining (red in B). Note the abundance and large size of H3K9me3 foci in cushion tissue mesenchyme (A) and myoblasts (B) of the OFT walls. (C) Double‐labeling for H3K9me3 (green) and β‐galactosidase (red) illustrating the strong expression of the senescence characteristic enzyme in the H3K9me3 enriched cells. (D–G) Double‐labeling for H3K9me3 (green) and TUNEL (red) illustrating a tentative sequence of cell degeneration from senescence to apoptosis. Characteristic cushion tissue senescent cell lacking TUNEL positivity (D); incipient TUNEL staining and H3K9me3 foci collapse in (E) and (F); and broad co‐localization of labeling in the latest stages of degeneration in (G). Scale bars: 10 μm.

The correlation of cell senescence with apoptosis was next analyzed at cell level in carefully dissociated tissue samples. Cell senescence is characterized by transcriptionally inactive heterochromatin DNA foci termed ‘senescence‐associated heterochromatic foci’ (SAHF), containing abundant Histone 3 trimethylated at lysine 9 (H3K9me3; Yu et al. 2018; Lee & Schmitt, 2019; Narita et al. 2003). Therefore, we combined the detection of H3K9me3 and beta‐galactosidase immunolabeling as complementary markers for cell senescence. As expected, both undifferentiated cushion cells and differentiating myocardial cells showed large foci of H3K9me3 in the areas of senescence (Fig. 4A,B). Beta‐galactosidase immunolabeling was abundant within the cytoplasm of H3K9me3 enriched cells (Fig. 4C). Furthermore, as shown in Fig. 4D–G, TUNEL assay in combination with H3K9me3 immunolabeling was suggestive of a sequence of degeneration events starting by cell senescence and progressing to apoptosis via activation of caspase‐dependent DNase.

### Transcriptional characterization of senescence‐associated secretory phenotype (SASP)

**Figure 5:**
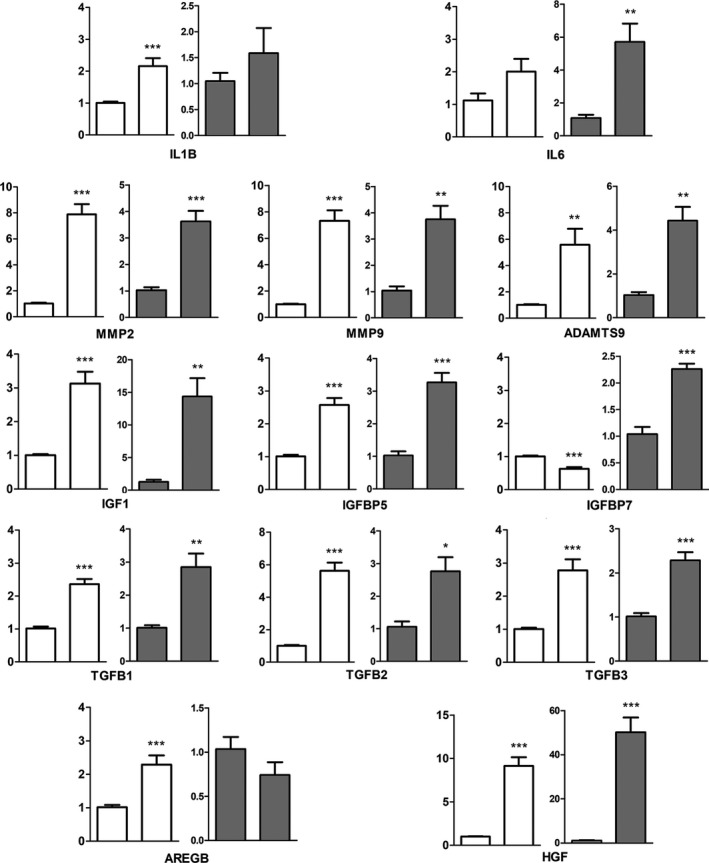
Charts showing the expression levels of a panel of SASP components in the remodeling OFT at stage 30HH (right bars in all charts), in comparison with adjacent non‐senescent heart wall of the same hearts (left bars in white charts) or with the wall of 23HH outflow tracts preceding the onset of senescence (left bars in gray charts). ***P < 0.001; **P < 0.01; *P < 0.05 vs. control.

To characterize the senescence‐associated secretory phenotype (SASP) at transcriptional level, we performed qPCR analysis of a panel of SASP components in fragments of the OFT tract isolated from embryos at 6 i.d. and 6.5 i.d. (29HH and 30HH). Some of these components are well known regulators of heart development, e.g. transforming growth factor β (TGFβs) members (Molin et al. 2003), but they are also well‐characterized SASP components in many other systems (Kuilman & Peeper, 2009). Comparative evaluation of their expression level values was made using two neutral non‐senescent regions: the wall of the ventricles subjacent to the OFT of the same hearts, and samples from the OFT of hearts of 4 i.d. embryos (23HH) that precede within 24 h the onset of senescence. As shown in Fig. 5, we identified intensified expression of secreted factors characteristic of the senescent secretome, including proinflammatory cytokines (*IL‐1b*,* IL‐6*); growth factors (*IGF1*;* IGFBP5*;* HGF*;* AREGB*;* TGF*β*s*); and tissue remodeling factors (*Adamts‐9*,* MMP2*,* MMP9*). IGFBP7 was only upregulated in comparison with the early OFT samples.

### Expression of cell cycle regulators

**Figure 6:**
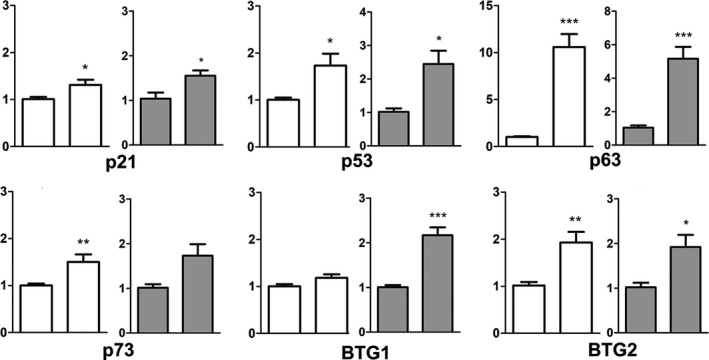
Charts showing the expression levels of a panel of cell proliferation regulators in the remodeling OFT at stage 30HH (right bars in all charts), in comparison with adjacent non‐senescent heart wall of the same hearts (left bars in white charts) or with the wall of 23HH outflow tracts preceding the onset of senescence (left bars in gray charts). ***P < 0.001; **P < 0.01; *P < 0.05 vs. control.

The expression of the most characteristic antiproliferative factors associated with cell senescence (see Czarkwiani & Yun, 2018) was monitored by qPCR using the same samples as above. As observed in other models of developmental senescence, *p21*,* p53*,* p63*, and *p73* were expressed in the OFT at significantly higher levels than in the non‐senescent samples (Fig. 6). The expression differences were particularly high for *p63*, which is a characteristic member of the *p53* family of tumor suppressor genes. We also observed a twofold increase in the expression level of *Btg2*, which is a tumor suppressor gene able to trigger senescence in the limb embryonic mesoderm (Lorda‐Diez et al. 2015).

### The developing mouse heart also presents areas of senescence

**Figure 7:**
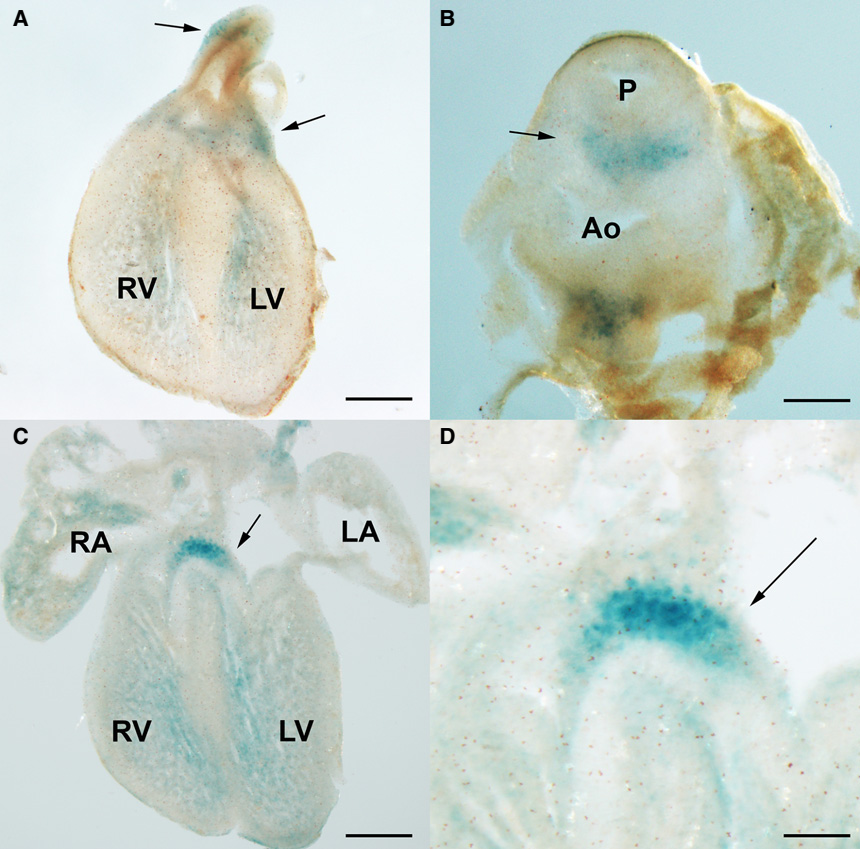
Areas of cell senescence in the developing mouse embryo heart at day 13.5 p.c. detected by SA‐β‐galactosidase activity staining. (A,B) Frontal (A) and transverse (B) sections showing SA‐β‐Gal staining around the outflow tract (arrows; A) and in the aorticopulmonary septum (arrows in B). (C,D) Low magnification (C) and detailed (D) views of the area of senescence in the auriculo‐ventricular region of the heart (C). Note intense labeling in the atrioventricular septum (arrow in C and D) and more scattered labeling in the subendocardial region of both ventricles. P, pulmonary artery; Ao, aorta; RA, right atrium; LA, left atrium; LV, left ventricle; RV, right ventricle: Scale bars: (A,C) 500 μm; (B) 250 μm, (D) 200 μm.

Considering the reported difference in the intensity of apoptosis between chick and mouse hearts, we also investigated the pattern of senescence in mouse embryo hearts. The study of zones positive for SA‐β‐Gal activity in the embryonic mouse heart was restricted to the period 12/14.5 days p.c. We found important areas of SA‐β‐gal staining in the OFT, the aorticopulmonary septum, the AV septum and the subendocardial myocardium. Except for the subendocardial myocardium, where staining was almost undetectable in the chicken, the above‐mentioned areas showed weaker intensity than found in chicken. As in the chick, the most intense positivity was observed in the OFT (arrow in Fig. 7A), especially at the aorticopulmonary septum (Fig. 7B). Cell senescence staining in the AV septum was intense (Fig. 7C,D), appearing in 12.5 p.c. embryos and remaining intense in 14.5 p.c. embryos.

The subendocardial positivity constitutes a differential feature in relation to the chick embryo. Labeling appeared widespread within the myocardium undergoing trabeculation, establishing a well‐defined borderline with the compact myocardium (Fig. 7A,C and data not shown).

### Increased lysosomal content and DNA damage in the zones of cell senescence and apoptosis

**Figure 8:**
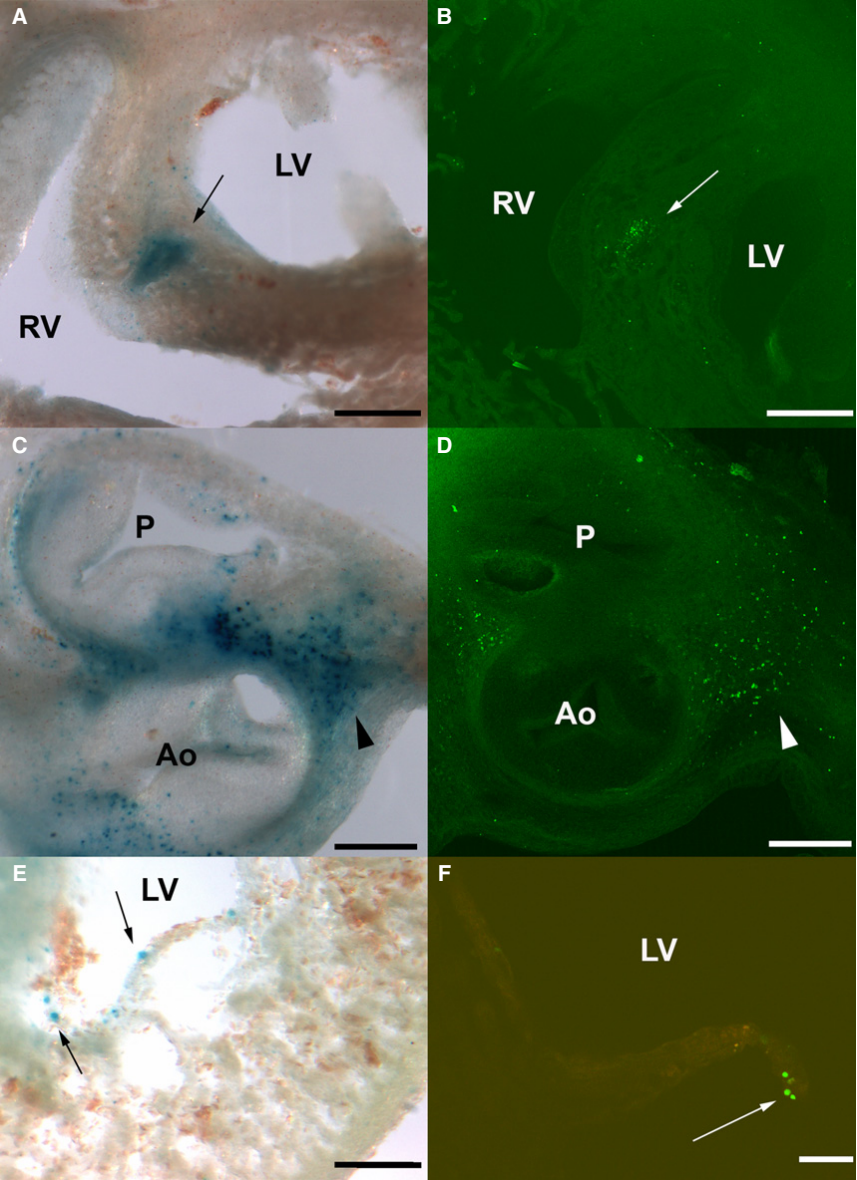
Correlation between TUNEL‐positive foci and the areas of SA‐β‐gal activity in the chick embryonic heart. (A–F) Correlative sections of the developing 33HH chicken heart stained for cell senescence by SA‐β‐Gal (A,C,E) and cell death by TUNEL (B,D,F). (A,B) Transverse sections at the level of the upper part of the interventricular septum showing overlapping senescent (A) and apoptotic (B) regions. (C,D) Transverse sections through the zona of formation of the arterial semilunar valves showing overlapping senescent (C) and apoptotic (D) foci. (E,F) Detailed views of the ventricular walls where scarce, isolated SA‐β‐Gal‐positive and TUNEL‐positive cells are often detected (arrows). P, pulmonary artery; Ao, aorta; RV, right ventricle; LV, Left ventricle. Scale bars: (A,B) 400 μm, (C,D) 300 μm, (E,F) 150 μm.

The areas of programmed apoptosis in the developing heart have been described in detail by different researchers (Pexieder, 1975; Hurle & Ojeda, 1979; Cheng et al. 2002). As shown in Fig. 8, the pattern of cell death identified by TUNEL labeling coincided with the areas of cell senescence that were detectable by SA‐β‐Gal labeling. Figure 8A,B shows the coincident labeling of cell senescence and TUNEL in the IVS. A similar coincidence is also appreciated in the OFT (Fig. 8C,D). TUNEL‐positive cells detected in the ventricular walls were also identified for SA‐β‐Gal (Fig. 8E,F).

**Figure 9:**
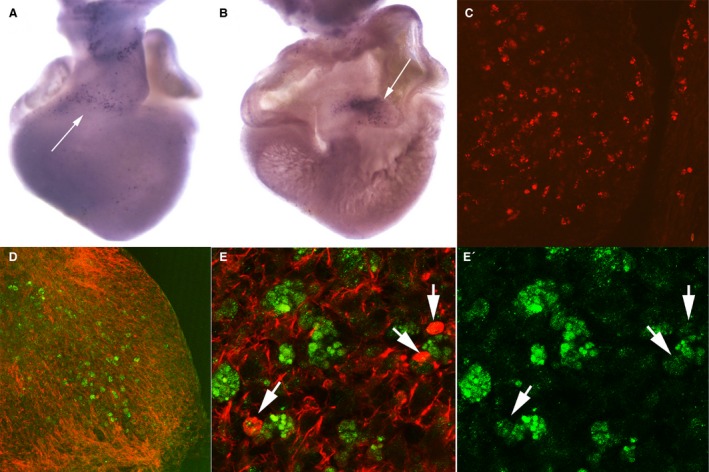
Lysosomal activity in the areas of cell senescence of the developing chick heart. (A,B) *In situ* hybridization in the developing chick heart at stage 29HH, showing expression of the *Cathepsin D* gene in the outflow tract (A) and atrioventricular septum (B). Comparison of the expression of *Cathepsin D* with the pattern of SA‐β‐Gal activity shown in Fig. 1A. (C) Transverse section of the outflow tract at stage 29HH, showing intense Cathepsin D immunolabeling (red) in the cushion tissue. (D,E) 29HH chicken heart sections showing β‐galactosidase immunolabeling (green) in combination with phalloidin staining (red) for actin. (E,E′) Detailed images showing β‐galactosidase positivity (green) in cells with actin clumps (red) characteristic of dying cells (arrows).

*Cathepsin D* expression correlates with areas of cell death in the developing heart (Fig. 9A–C) and is expressed at the level of the OFT (arrow in Fig. 9A), including the endocardial cushions (Fig. 9C) and the AV septum (arrow in Fig. 9B). In the same areas, β‐galactosidase was notably expressed at protein level (Fig. 9D,E′) in correlation with the SA‐β‐Gal activity described above. It is noticeable that within the areas where the expression of cathepsin D and β‐galactosidase was high, there were abundant cells with clear signs of degeneration, identifiable by the disorganization of the actin cytoskeleton forming abnormal clumps of actin (arrows in Fig. 9E,E′). This is a characteristic sign of dying cells (Montero et al. 2016) that supports an apoptotic fate for cells in the areas of senescence.

**Figure 10:**
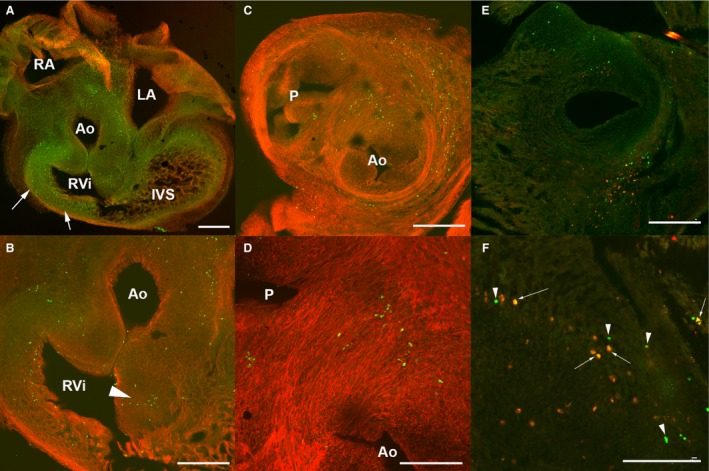
DNA damage in the developing chick heart detected by γH2AX immunolabeling. (A–D) Stage 31HH chicken heart sections immunolabeled for phosphorylated H2AX (γH2AX; green) and counterstained with phalloidin (red). (A,B) Frontal section of the heart showing abundant γH2AX‐positive cells among the outflow tract and especially the right ventricular wall (arrows in A). (B) Detailed view of (A) to better visualize an area of γH2AX‐positive cells in the lowest region of the cushion tissue (arrowhead in B). (C,D) Transverse section through the outflow tract showing abundant cells positive for γH2AX. (D) Detailed view of (C) showing distribution of γH2AX‐positive cells in the aortopulmonary septum. (E,F) Heart section at the OFT level of a stage 30HH showing γH2AX (green) and TUNEL (red) double‐staining. (F) Detailed view. Note abundance of only green‐labeled cells (arrowheads) and double‐labeled cells (arrows). P, pulmonary artery; Ao, aorta; RA, right atrium; LA, left atrium; RVI, right ventricle infundibulum. Scale bars: (A–C,E) 300 μm, (D,F) 150 μm.

We recently observed that in the developing limb, DNA damage prefigures the areas of cell death detected by the TUNEL assay (Montero et al. 2016). We employed the detection of γH2AX to explore whether cell death and cell senescence in the embryonic heart is also accompanied by DNA damage. The initial response of cells to DNA damage is activation at the DNA break region of substrates that contribute to repairing the ruptures. During this process, the histone variant H2AX is phosphorylated at serine 139 (γH2AX) in the damaged region (Redon et al. 2011), which allows the additional recruitment of DDR mediators. Here we used a γH2AX antibody that maps to a region surrounding phosphorylated serine 139 of human histone H2AX for immunolabeling. Figure 10 illustrates the presence of γH2AX in cells of the developing heart; γH2AX cells are specifically distributed in the same regions of cell senescence and cell death. In Fig. 10A,B, γH2AX staining is detected in cell nuclei of the developing OFT and the ventral RV myocardium (arrows in Fig. 10A). Labeling is also detected in cells at the level of the AV septum in the AV cushions (arrowhead in Fig. 10B). Abundant positive cells are detected along the whole OFT, especially at the level of the aorticopulmonary septum (Fig. 10C,D). As suggested in a different embryonic model of degenerative events (Lorda‐Diez et al. 2015; Montero et al. 2016), we hypothesized that also in the developing heart, DNA damage may precede senescence and the caspase‐mediated apoptosis. In agreement, as would be expected if this were so, in stage 30HH heart sections double‐labeled for γH2AX and TUNEL, almost 40% of the degenerating cells were positive only for γH2AX labeling (Fig. 10E,F; arrowheads). As an exception, in a very reduced number of cells that were only TUNEL‐positive, the remaining degenerating cells were double‐positive for γH2AX and TUNEL (Fig. 10E,F; see arrows).

## Discussion

Cell death by apoptosis is believed to play a major role in vertebrate heart development (Pexieder, 1975; Fisher et al. 2000; Van den Hoff et al. 2000; Schaefer et al. 2004). However, its physiological relevance and integration with other remodeling mechanisms are poorly understood. Among the functions proposed for cell death in the embryonic heart are the following: fusion of the paired heart primordia (Ojeda & Hurle, 1975); remodeling and septation of the outflow tract (Hurle et al. 1977; Hurle & Ojeda, 1979; Rothenberg et al. 2003; Sugishita et al. 2004; Okamoto et al. 2010); sculpting of the conduction system and isolation of auricles from ventricles (Vicente‐Steijn et al. 2018); excavation of the cusps of the semilunar valve primordia (Hurle et al. 1980); formation of the coronary artery orifices in the aortic wall (Velkey & Bernanke, 2001); and control of heart size (Zhou et al. 2015).

We now know that the biological significance of apoptosis in embryonic and tumoral degenerative processes is more complex than initially thought (Doherty & Baehrecke, 2018). In fact, in addition to the removal of tissues, apoptosis may also play a role promoting compensatory overgrowth (Hurle, 2014). The role of caspases as executors of apoptosis was considered central, as they were discovered early in *C. elegans*. However, programmed tissue regression in more complex organisms is not always impaired when caspase function is abrogated (Kuida et al. 1998; Chautan et al. 1999; 8; Montero & Hurle, 2010). Cardiac caspase‐deficient phenotypes are particularly interesting in this context. Mutants for caspases, or their regulators, revealed developmental heart defects independent of cell death (Varfolomeev et al. 1998; Yeh et al. 1998, 2000). The heart morphology in mice with heart‐targeted double KO for caspases 3 and 7 is particularly illustrative (Cardona et al. 2015). Contrary to expectations, the resulting phenotype is a smaller heart with decreased cell numbers. Defects were due to altered cardiomyocyte DNA replication and cell cycle progression (Cardona et al. 2015). *Caspase 8*‐deficient mice also bear developmental abnormalities, including thinner ventricular walls and disorganized trabeculae. In this case, the defects appeared related to myocyte differentiation rather than anomalous patterns of cell death (Varfolomeev et al. 1998). Indeed, in spite of the high expression level in the developing ventricles of apoptotic effectors such as FAS/FASL or caspases (Varfolomeev et al. 1998; Sharma et al. 2004; Cardona et al. 2015), cell death intensity is low in the ventricular walls (Sharma et al. 2004). Thus, caspases seem to play other roles in addition to promoting degenerative processes, and their knockdown phenotypes do not fully fit with impairment of cell death. Together these findings indicate that the dying program in the embryonic heart involves additional, and most likely redundant or complementary, caspase‐independent mechanisms. Our findings show that cell senescence, increased lysosomal content and DNA damage are degenerating features that accompany programmed cell death during heart morphogenesis.

Senescence is a cellular response to stress observed both in normal and tumoral tissues. It was considered an irreversible cell cycle arrest associated with aging and carcinogenesis. More recently, cell senescence has been associated with tissue repair and embryonic development (Czarkwiani & Yun, 2018). In these systems, cell senescence appears to be a transitory event associated with tissue removal and with the modulation of cell differentiation in neighboring tissues (Muñoz‐Espin et al. 2013; Storer et al. 2013; Demaria et al. 2014; Lorda‐Diez et al. 2015). Our findings reveal well‐defined areas of cell senescence that overlap with the areas of apoptosis present in the developing heart of chick and mouse embryos, including the developing outflow tract, the atrioventricular orifices and the closure of the interventricular foramen. Consistent with the reduced distribution and intensity of the apoptotic areas in the mouse in comparison with those of the chick, as reported in previous studies, the areas of senescence‐associated SA‐β‐Gal positivity in the mouse heart are much reduced compared with those found in the chick. This similitude in the patterns of apoptosis and senescence suggests complementary functions for both processes and the occurrence of common upstream triggering signals. DNA damage often accompanies cell senescence (Hernandez‐Segura et al. 2018) and may be a triggering factor for senescence and apoptosis (d'Adda di Fagagna et al. 2003; Herbig et al. 2004; Rodier et al. 2009; Rossiello et al. 2014). A dynamic feedback loop has been proposed in which maintaining the DNA repair response generates reactive oxygen species, which in turn actively promote a stabilized senescent state. Here, we show that cells positive for γH2AX immunolabeling were abundant within the zones of cell senescence. γH2AX is an early marker of DNA breaks that helps to recruit DNA repair factors. Indeed, the presence of abundant γH2AX foci has been described in the nuclei of cells initiating the senescence program (Chen & Ozanne, 2006). γH2AX favors senescence by promoting both senescence‐associated growth arrest and secretory phenotype (Rodier et al. 2011). Together these findings indicate that heart remodeling in the embryo involves joint cooperation of various degenerative routes that likely respond to common signals.

A central function of senescent cells is the production of a number of secreted factors known as the senescence‐associated secretory phenotype, which covers a wide range of biological activities able to modify the function of non‐senescent neighbor cells (Bernard, 2018). Although senescent processes share many SASP components, differences occur depending on the cell lineage and the senescence inducer (Coppé et al. 2010). Our transcriptional analysis identified high expression levels of growth factors (*TGF*β*‐1*,* 2* and ‐ *3*;* HGF*;* AREGB*;* IGF1*; and *IGFBP5*), a proinflammatory cytokine (*IL‐1b*), and metalloproteinases (*MMP2*;* MMP9*;* ADAMTS9*), in the outflow tract of the chick embryo heart. These factors are characteristic members of the SASP in oncogenic, replicative and developmental senescence models (Lorda‐Diez et al. 2015; Sapieha & Mallette, 2018).

The establishment of the senescent state is associated with the expression of antiproliferative factors. Although there are variations among distinct models of senescent cells in antiproliferative mediators (Czarkwiani & Yun, 2018), *p21* and the family of *p53* tumor suppressor genes are the most characteristic factors associated with senescence. Our analysis in chick embryonic samples identified *p63* as the highest expressed member of the *p53* gene family. In addition, we also detected high expression levels of *Btg2*, a representative member of the *Btg/tob* family of tumor suppressor genes. *Btg2* shows a regulated expression in the areas of interdigital cell death, and its overexpression causes cell senescence in the developing limb (Lorda‐Diez et al. 2015).

Considering the potential morphogenetic relevance of cell senescence, we showed a significant difference in the pattern of senescence between chick and mouse hearts in the subendocardial region of the ventricles. The endocardial surface of the ventricles was intensely trabeculated in both chick and mouse hearts, but the trabeculation process showed significant differences (Sedmera et al. 1997; Captur et al. 2016). Whether the abundance of myocardial cells positive for SA‐β‐Gal in the subendocardial region of the embryonic mice hearts is representative of mechanistic differences in sculpturing the trabeculae or they reflect biomechanical or bioelectrical differences of ventricular trabeculae between both species remains to be clarified.

## Conflict of interest

The authors declare no conflict of interest.

## Author contributions

CILD, MESM, JMH, JAGP and JAM conceived and designed the experiments. CILD, MESM, CSF, JAM and JMH performed the experiments. CILD, MESM, JMH and JAM analyzed the data. CILD, JAM and JMH wrote the manuscript.
